# Editorial: Multi-omics technology: revealing the pathogenesis of diseases and the mechanism of drug efficacy of major diseases such as nutritional metabolism disorders and mental disorders

**DOI:** 10.3389/fphar.2025.1586223

**Published:** 2025-03-17

**Authors:** Wenzhi Hao, Tianhao Liu, Xiliang Du, Gabriel Tao

**Affiliations:** ^1^ School of Traditional Chinese Medicine, Jinan University, Guangzhou, China; ^2^ Affiliated Hospital of Jiangnan University, Wuxi, China; ^3^ Jilin University, Changchun, China; ^4^ Merck Sharp & Dohme Corp (United States), West Point, PA, United States

**Keywords:** nutritional and metabolic disorders, mental disorders, multi-omics technology, natural products, depression

With the changes in the living patterns and dietary patterns of modern people, the incidence of nutritional and metabolic disorders as well as mental disorders is gradually increasing, and the two interact with each other. It is of great significance to explore the pathologic mechanism of nutritional and metabolic disorders as well as mental disorders and to develop drugs to intervene in the pathogenesis for the understanding and clinical treatment of diseases. With the development and progress of science and technology, high-throughput sequencing such as proteomics, metabolomics, transcriptomics, and microbiomics plays an important role in revealing the pathogenesis of diseases and the mechanism of drug efficacy. In fact, multi-omics technology has now become an important tool for understanding the pathogenesis of diseases and elucidating the efficacy mechanisms of drugs. The following discussion will center on the topics of the 9 most recent important research in our area, deepening our understanding of the role of multi-omics technology in understanding the mechanisms of diseases and exploring pharmacological effects.

Gastric cancer is one of the commonest gastrointestinal malignant tumors worldwide, Weizhuan’an prescription is the classic Chinese medicine compound for treatingthe precancerous lesions of gastric cancer. By using 16S rDNA amplicon sequencing, researchers have confirmed that Weizhuan’an prescription can treat rats with PLGC by regulating gastric mucosal microflora and inflammatory factors, providing more references for clinical practice (Lu et al.). Metabolic dysfunction-associated steatotic liver disease (MASLD) is the most common metabolic syndrome and chronic liver disease worldwide, Lingguizhugan (LGZG) decoction is a classic formula treating various liver diseases such as MASLD. By using serum untargeted metabolomics, gut microbiota, bile acid metabolism, immunohistochemistry, and Western blotting analyses, researchers have found that modulating microbiota–BA–FXR/TGR5 signaling pathway may be a potential mechanism of action of LGZG oral solution for the treatment of MASLD (Wang et al.). Heart failure (HF) is a chronic condition that progressively worsens and continues to be a major financial burden and public health concern. Shenfu injection (SFI) is one of the representative prescriptions of the warming Yang method, and to be applied extensively in the treatment of heart failure with remarkable curative effects. By utilizing a comprehensive methodology, incorporating various techniques such as echocardiography, protein chip detection, histopathology, 16S rDNA sequencing, and metabolomics, the researchers have found that SFI improves ISO-induced heart failure by modulating cometabolism and regulating the TMAO-inflammation axis (Li et al.). Alzheimer’s disease (AD) and Parkinson’s disease (PD) are the two most common neurodegenerative disorders that have a significant impact on the aging population worldwide. Yangxue oral liquid (YXKFY) is composed of four traditional Chinese medicines. Through a synergistic approach combining transcriptome and proteome analyses with machine learning, the researchers have innovatively identified 17 and 3 hypoxia-associated biomarkers for AD and PD, respectively. Furthermore, they have elucidated the neuroprotective mechanisms of YXKFY, highlighting its antioxidant properties (Chen et al.). High concentrations of nonesterified fatty acids (NEFA) is the key of characteristic of fatty liver in dairy cows. Through the lipidomic approach and molecular biology techniques, the researchers have found that high concentration of NEFA is lipotoxic to cells, promoting lipid accumulation (Fan et al.). Xylene exposure is known to induce toxicity in hematopoietic stem and progenitor cells, leading to bone marrow suppression and potential leukemogenesis. Coniferyl ferulate is a phenolic acid compound abundant in umbelliferae plants with multiple pharmacological activities. By using single-cell RNA sequencing, the researchers have identified CF and Mgst2 as potential therapeutic targets for alleviating xylene-induced hematotoxicity (Yin et al.). Spatial metabolomics is an emerging technology that integrates mass spectrometry imaging (MSI) with metabolomics, offering a novel visual perspective for traditional metabolomics analysis. The researchers have summarized the latest progress and challenges of applying spatial metabolomics to the study of mental disorders and traditional Chinese medicine (Lei et al.). Angelica sinensis, a traditional Chinese herbal medicine and food, which has a long history of clinical application. The researchers have established a theoretical foundation for future studies on the structure, mechanism, and clinical use of Angelica sinensis (Ren et al.). Hypertension is a common disease. By integrating the results of multiple omics studies, the researchers found that the use of TCM to treat hypertension by regulating the intestinal flora is a promising therapeutic strategy (Chen et al.).

Existing evidence suggests that the multi-omics technology has now become an important tool for understanding the pathogenesis of diseases and elucidating the efficacy mechanisms of drugs. Our research theme mainly reveals that multi-omics technology has been widely used to explore the pathological mechanism of nutritional and metabolic disorders and mental disorders, and explore the therapeutic mechanism of TCM compound or monomer natural active ingredients for diseases. In summary, the studies discussed in this Research Topic highlight the important role of the multi-omics technology in various health conditions and the potential for TCM to modulate nutritional and metabolic disorders as well as mental disorders for therapeutic purposes.

